# On the Eau Medicinale d'Huson

**Published:** 1813-01

**Authors:** 


*To the Editors of the Medical and Physical Journal.*

GENTLEMEN,

AS private correspondence may sometimes tend to public utility, I am induced to send for insertion in your valuable Journal a letter received from Paris on the subject of the so-much-talked-of *Eau Médicinale*.

A friend, having occasion for a quantity of this medicine, applied to a physician at Paris to procure it, and received the following answer. T. C.

" *Quelle que fut, Monsieur, l'occasion de faire quelque chose qui vous fut agreable. J'ai saisie avec plaisir et empressement; J'ai fait, mais sans succès, toutes les recherches possibles pour découvrir l'auteur et le dépot de l'Eau Médicinale d'Huson que vous desirez pour votre ami gouteux. Soit discredit bien motive du remede, soit par suite des dispositions du gouvernement sur les remedes secrets, l'auteur à disparu, et on ne le trouve ni lui, ni sa drogue, aux differens domiciles dont il avoit successivement donné l'addresse. Je dois vous ajouter, d' après les informations que l'intérêt que vous y prenez m'a engagé à faire, que cette eau, pretenduë antigouteuse, est un purgatif acre et très violent, qui a pu être utile à un petit nombre*

*nombre de malades pituiteux; mais qu'il a produit les plus mauvais effets chez la plupart, de ceux qui en ont usé.—J'ai cru ne devoir pas différer à vous communiquer ces instructions pour vous prouver mon zele; ce qui ne m'empechera pas de continuer mes recherches pour vous donner, s'il est possible, plus ample satisfaction.*

*Votre très humble et très obéissant serviteur,*

*(Signé)* MENURET, Med.

TRANSLATION.

Sir,—I eagerly embrace the opportunity of communicating any thing which may afford you pleasure. I have made, but without success, every possible inquiry to discover the author and the depôt of the *Eau Medicinale d'Huson,* which you desire for your gouty friend.—Whether from the well-founded disrepute of the medicine, or from the intentions of government against unknown medicines, the author has disappeared, and neither he nor his medicine is to be found in the various houses from which he has successively announced them. I ought to add that, (after the inquiries which, from the interest you take in them, I have been induced to make,) this pretended gout medicine is a sharp and violent purgative, which has been of use to some few phlegmatic persons, but has produced the most pernicious effects to the greater part of those who have used it. I have thought it right not to delay communicating this information, in order to prove my readiness to serve you, and nothing shall hinder me from continuing my inquiries, to give you (if possible) more ample satisfaction.

Your very humble and obedient servant,

(Signed) MENURET, Physician.

